# Supplementary material for: Association between oral microbiome and seven types of cancers in East Asian population: a two-sample Mendelian randomization analysis
Source: Front Mol Biosci. 2023 Nov 21;10:1327893. doi: 10.3389/fmolb.2023.1327893 (PMC10702768; doi:10.3389/fmolb.2023.1327893)
Supplement: Supplementary file 1 [file DataSheet1.ZIP › Supplementary Materials/MR plots for tongue/tongue═╝/Ovarian cancer/pheno.3249_to_ovarian cancer_leave_one_out.pdf]

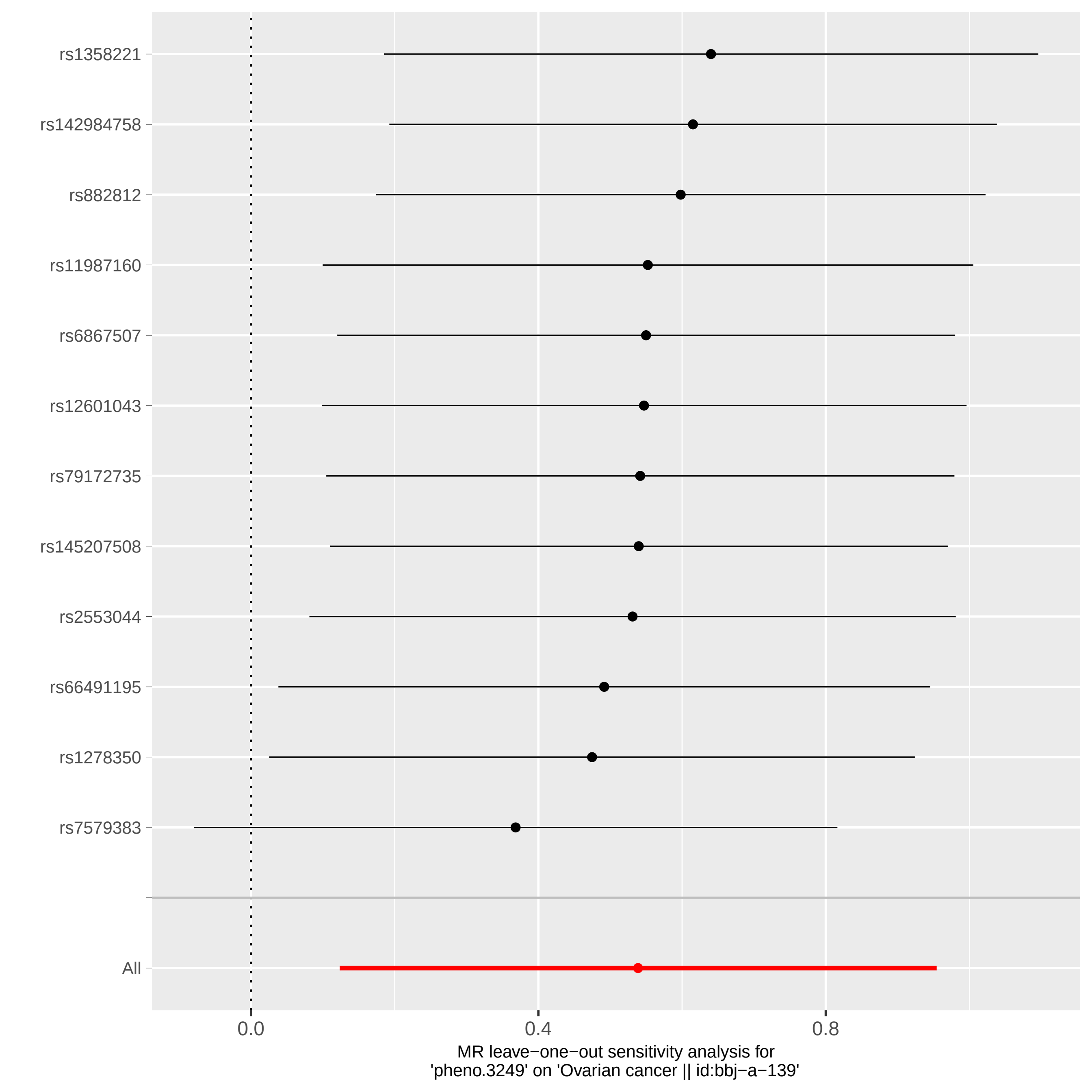
rs1358221
rs142984758
rs882812
rs11987160
rs6867507
rs12601043
rs79172735
rs145207508
rs2553044
rs66491195
rs1278350
rs7579383
All
0.0
0.4
0.8
MR leave−one−out sensitivity analysis for
'pheno.3249' on 'Ovarian cancer || id:bbj−a−139'
